# The Fungicide Ipconazole Can Activate Mediators of Cellular Damage in Rat Brain Regions

**DOI:** 10.3390/toxics12090638

**Published:** 2024-08-31

**Authors:** Carlos Villaorduña, Luis Barrios-Arpi, Boris Lira-Mejía, Mariella Ramos-Gonzalez, Olger Ramos-Coaguila, Luis Inostroza-Ruiz, Alejandro Romero, José-Luis Rodríguez

**Affiliations:** 1 Animal Physiology Laboratory, Faculty of Veterinary Medicine, Major National University of San Marcos, Lima 15021, Peru; 2 Zootecnia an Animal Production Laboratory, Faculty of Veterinary Medicine, Major National University of San Marcos, Lima 15021, Peru; 3 Toxicology Laboratory, Faculty of Pharmacy and Biochemistry, Major National University of San Marcos, Lima 15021, Peru; 4 Department of Pharmacology and Toxicology, Faculty of Veterinary Medicine, Complutense University of Madrid, 28040 Madrid, Spain

**Keywords:** neurotoxicity, ipconazole, rat brain regions, oxidative stress, cell death, inflammasome

## Abstract

This study aimed to investigate the toxicity of the fungicide ipconazole on oxidative status, cell death and inflammasome complex activation in the hypothalamus, cerebral cortex, striatum and hippocampus of rats. Female albino rats were randomly divided into a control group and four groups treated with ipconazole at doses of 1, 5, 10 and 20 mg/kg b.w., administered for six days. Ipconazole significantly increased MDA and ROS levels in all brain regions studied, while reducing catalase enzyme activity. The molecular expression of cell death-related genes (AKT1, APAF1, BNIP3, CASP3 and BAX) and the inflammasome complex (CASP1, IL1β, IL6, NLRP3, NFĸB and TNFα) was also assessed, showing increased expression in at least one brain region. The findings demonstrate that ipconazole induces central nervous system toxicity in mammals, highlighting its potential role as a risk factor in the development of neurodegenerative disorders in individuals exposed to this contaminant.

## 1. Introduction

Agrochemical pesticides are widely used to control pests affecting crops, with their usage increasing every year [1]. Among the most commonly used pesticides in agriculture are fungicides, which are compounds that target fungi affecting crops. These fungicides can enter ecosystems as contaminants through various pathways, producing toxic effects on a wide variety of non-target organisms [2].

One of the most widely used classes of agricultural fungicides worldwide is the triazoles. These fungicides are now recognized as environmental contaminants in both aquatic and terrestrial environments, even reaching drinking water [3]. The physicochemical properties of agricultural triazoles indicate that they have a low molecular weight and are moderately lipophilic, allowing them to accumulate on organic surfaces [2,4]. This accumulation over long periods results in continuous low-dose exposure for animals and humans, negatively impacting ecosystems and the health of living organisms within them [5].

Several in vivo and in vitro studies have reported the toxicity of triazoles in non-target organisms [6,7,8,9,10,11,12,13,14,15,16,17,18,19]. For example, the triazole propiconazole has been shown in vivo to alter reproductive physiology, hepatic CYP enzyme activity, antioxidant enzyme activity and hypothalamic–pituitary–thyroid axis activity [6,7,8,9]. In vitro studies have demonstrated that it can induce inflammation in liver cells [10]. Additionally, penconazole has been found to inhibit antioxidant enzymes and alter nervous system development [11,12]. The triazole tebuconazole causes gene toxicity, reproductive and sensorimotor impairment, and oxidative stress, among other toxic effects, in both in vivo and in vitro studies [13,14,15,16]. The triazole ipconazole, the subject of the present study, has not been widely reported as toxic, but it has been shown to reduce sensorimotor activity and cause toxicity in neuronal and endothelial cells [17,18,19].

Ipconazole is an agricultural pesticide that acts by inhibiting ergosterol biosynthesis in fungi and was synthesized in 1986 especially for use on rice seeds [20]. Currently, ipconazole is used to control a number of diseases caused by various fungi, such as *Rhizoctonia solani*, *Fusarium fujikuroi* and *F. oxysporum*, in more than 50 crops, including maize, cotton and cereals. However, misuse and abuse of this fungicide is leading to resistance in target pathogens [21] or levels that exceed maximum residue limits (MRLs) [22] and could cause health problems in operators, consumers, production animals and other non-target organisms [23]. The Commission of the European Union has revoked the marketing of ipconazole as a plant protection active substance because it presents a high long-term risk to birds and is classified as a category 1B reproductive toxicant, and also because of uncertainty about its toxicity to humans [24].

The toxicity induced by the fungicide ipconazole in non-target organisms, including humans, is a latent concern that needs to be addressed. For this reason, the present study developed several assays (ROS production, MDA levels, catalase activity and molecular assays) to evaluate ipconazole fungicide-induced toxicity in four brain regions (hypothalamus, cerebral cortex, striatum and hippocampus) of rats.

## 2. Materials and Methods

### 2.1. Chemicals

Antibiotics (gentamicin, streptomycin and penicillin G); fluorescent probes 4-amino-5-methylamino-2,7-difluorofluorescein-diacetate (DAF-FM-DA) and 2′,7′-dichlorofluorescin-diacetate (DCFH-DA); Dulbecco’s phosphate buffered saline; Dimethyl sulfoxide; and 4,6-Dihydroxy-2-mercaptopyrimidine were obtained from Sigma-Aldrich (Saint Louis, MO, USA). For molecular assay, NucleoSpin RNA-extraction, the kit for cDNA synthesis and MasterMix ICgreen were obtained from Cultek (Madrid, Spain). The catalase colorimetric activity assay kit was obtained from Invitrogen (Thermo Fisher Scientific, Waltham, MA, USA). All other chemicals were obtained from the usual commercial sources and were of the highest grade available.

### 2.2. Animals and Experimental Design

The study was conducted in accordance with the requirements of the Bioethics Committee of the Universidad Nacional Mayor de San Marcos, and in accordance with the international requirements of the 3Rs. Fifteen female albino rats (Peruvian National Institute of Health), weighing approximately 200 g each and 8 weeks old, were used. The animals were reared in polycarbonate cages with sawdust bedding; environmental conditions were controlled (22 ± 2 °C and 50 ± 10% relative humidity) with a 12 h light cycle. Feed and drinking water were supplied ad libitum.

Treated animals received a single daily oral administration of the fungicide ipconazole at doses of 1, 5, 10 and 20 mg/kg body weight (b.w.) for 6 consecutive days. In this study, the doses of ipconazole were 1/888, 1/177, 1/88 and 1/44 of the LD_50_ [25]. Animals in the control and ipconazole-treated groups were deprived of food for 6 h prior to oral administration of ipconazole but were allowed water ad libitum. Doses of ipconazole were dissolved in corn oil for subsequent oral administration at a maximum volume of 0.5 mL/rat. Control animals received only corn oil (vehicle).

Animals were euthanized by decapitation 24 h after the last administration, after which the brains were removed and rinsed with 0.9% (*w*/*v*) sodium chloride to remove traces of other tissues. Brain regions (hypothalamus, cerebral cortex, striatum and hippocampus) were dissected out cold (4 °C) and immediately frozen at −80 °C for further analysis.

### 2.3. Reactive Oxygen Species (ROS) Generation Assay

A total of 10 mg of each brain region was homogenized in a buffer containing 50 mM HEPES, 320 mM sucrose and protease inhibitors. Immediately, a total of 15 µL of homogenate (equivalent to 1 mg of brain region) was added to 80 µM (final concentration) of DCFH-DA (diluted in DMSO) in phosphate-buffered saline (PBS). The reaction was carried out in black 96-well plates at an incubation temperature of 37 °C for 30 min [26,27]. ROS production was recorded on a FLX800 fluorometer (BioTek, Winooski, VT, USA) with an excitation/emission wavelength (485 nm/528 nm). 

### 2.4. Determination of Malondialdehyde (MDA) Levels

In total, ≥10 mg of brain region was homogenized with lysis buffer containing sodium duodecyl sulphate (SDS). The homogenate was centrifuged at 13,000× *g* for 15 min at 4 °C. Then, 250 μL of the supernatant was vigorously mixed with 250 µL of thiobarbituric acid (0.07%) dissolved in sodium sulphate solution [28]. The mixture was incubated at 95 °C for 60 min and read spectrophotometrically at 532 nm (Agilent Technologies, Santa Clara, CA, USA).

### 2.5. Catalase Activity Assay

For this assay, the catalase colorimetric activity assay kit (Invitrogen, Thermo Fisher Scientific, MA, USA) was used according to the manufacturer’s guidelines. Briefly, ≥20 mg of brain region was homogenized with the kit-supplemented buffer at 4 °C, then the homogenate was centrifuged at 10,000× *g* for 15 min at 4 °C, and the supernatant was immediately processed. In a 96-well plate, 25 µL of the sample was mixed with 25 µL of hydrogen peroxide reagent for 30 min at room temperature, then 25 µL of horseradish peroxidase was added and incubated for 15 min at room temperature. Spectrophotometric reading was performed at 560 nm (Agilent Technologies, CA, USA), and optical density values were calculated from the standard curve fit to obtain the catalase activity in U/mL/mg tissue.

### 2.6. qPCR Analysis of Cell Death and Inflammasome Complex Biomarkers

Molecular expression of genes related to cell death and the inflammasome complex was performed using 3 kits: RNA extraction, retrotranscription (RT-PCR) and molecular amplification (qPCR). (1) Total RNA extraction was performed from ≥15 mg of brain region following the manufacturer’s specifications (NucleoSpin RNA Plus Kit, MACHEREY-NAGEL, Düren, Germany), and the concentration was measured in a nanospectrophotometer (Microdigital, Seoul, Republic of Korea) obtaining A260/A280 ratios between 1.9 and 2.1. (2) RT-PCR was performed on 400 ng of total RNA using the cDNA synthesis kit (PCRBiosystems, Wayne, PA, USA) under thermocycling parameters of 42 °C for 30 min and 85 °C for 10 min. (3) Gene amplification and quantification was performed using MasterMix ICgreen (Nippon Genetics, Duren, Germany) according to supplier specifications, and the thermocycling protocol used was 95 °C for 2 min, 40 cycles of 5 s at 95 °C and 30 s at 60 °C. This was performed with Bio-Rad CFX (BioRad, Hercules, CA, USA) [29]. The primers used in this study were specific for cell death (AKT1, APAF1, BNIP3, CASP3 and BAX) and the inflammasome complex (CASP1, IL1β, IL6, NLRP3, NFκB and TNFα) biomarkers (Supplementary Material S1).

### 2.7. Statistical Analysis

Data were analyzed using GraphPad Prism 8 statistical software(GRAPHPAD SOFTWARE, BOSTON, MA, USA). Results are presented as the percentage (%) or fold change compared to the control and expressed as the mean ± SEM per group. Significant differences between control and treated groups were determined using one-way ANOVA followed by Tukey’s post hoc test. Results were considered significant at * *p* < 0.05, ** *p* < 0.01 or *** *p* < 0.001.

## 3. Results

In the present study, we evaluated the effect of ipconazole at doses of 1, 5, 10 and 20 mg/kg b.w. during 6 days of treatment on the hypothalamus, cerebral cortex, striatum and hippocampus brain regions of albino rats. Briefly, we observed that ipconazole produced a deleterious effect in the rat brain, increasing the levels of ROS, MDA and genes related to cell death and the inflammasome complex; in addition, the levels of catalase enzyme activity were reduced by ipconazole. These results are described in detail below.

### 3.1. ROS Production 

**Figure 1:**
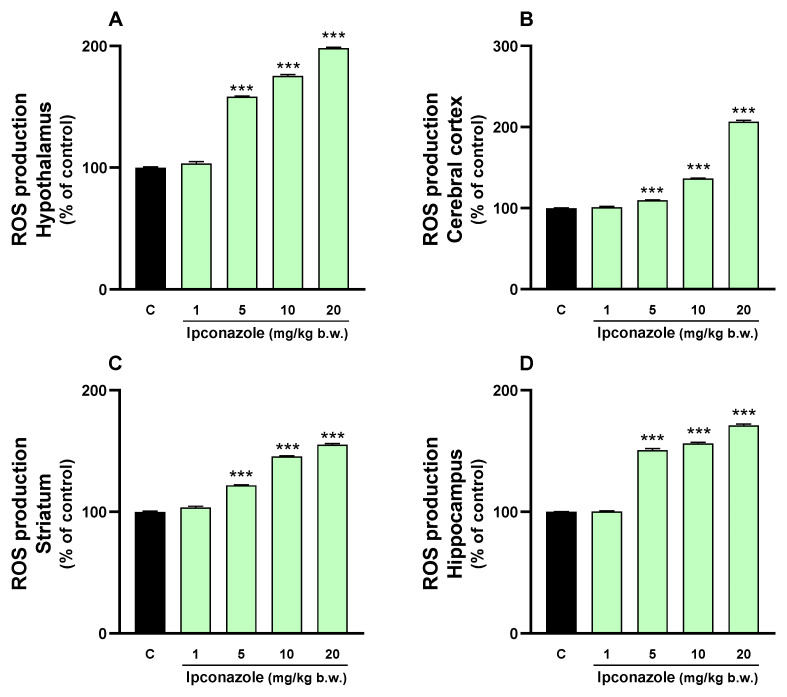
ROS production was measured in the (**A**) hypothalamus, (**B**) cerebral cortex, (**C**) striatum and (**D**) hippocampus of rat brains. Results are presented as a percentage of the control (mean ± SEM) based on four replicates per treatment. The significance between the treatments (green bar) and the control group (black bar) was determined by one-way ANOVA using Tukey’s post hoc test. *** *p* ≤ 0.001 indicates significant differences between the treatments versus the control group.

Oxidative stress can trigger multiple deleterious effects in cells, including damage to biomolecules, altered cellular responses, inflammatory processes and even cell death. In the present study, we evaluated ROS production induced by the fungicide ipconazole (1, 5, 10 and 20 mg/kg b.w.) in four regions of rat brains. Ipconazole significantly (*** *p* ≤ 0.001) increased ROS levels in a dose-dependent manner (5, 10 and 20 mg/kg b.w.) in the hypothalamus (by 58, 75 and 98%, respectively) (Figure 1A), cerebral cortex (by 10, 36 and 106%, respectively) (Figure 1B), striatum (by 21, 45 and 55%, respectively) (Figure 1C) and hippocampus (by 50, 56 and 71%, respectively) (Figure 1D).

### 3.2. MDA Levels

**Figure 2:**
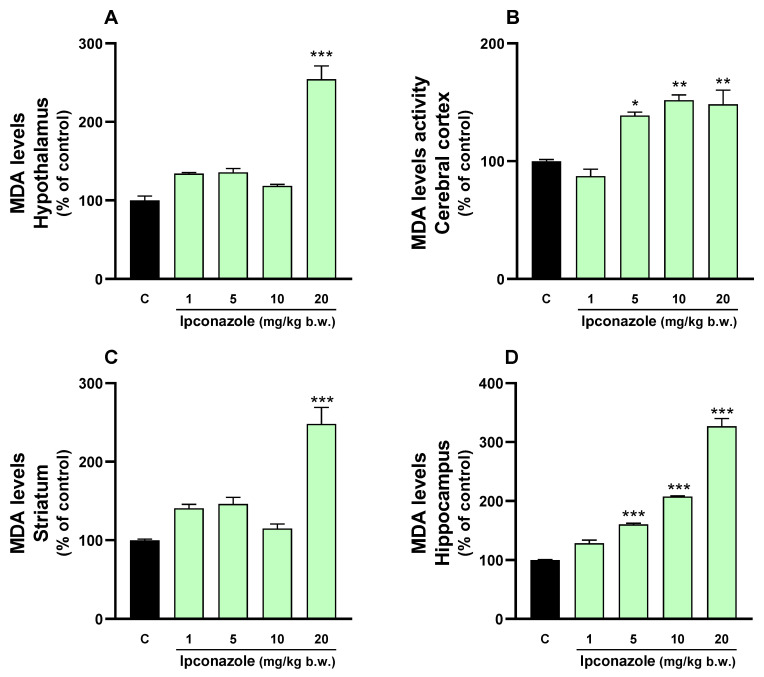
MDA levels were measured in the (**A**) hypothalamus, (**B**) cerebral cortex, (**C**) striatum and (**D**) hippocampus of rat brains. Results are presented as a percentage of the control (mean ± SEM) based on four replicates per treatment. The significance between the treatments (green bars) and the control group (black bar) was determined by one-way ANOVA using Tukey’s post hoc test. * *p* ≤ 0.05, ** *p* ≤ 0.01 and *** *p* ≤ 0.001 indicate significant differences between the treatments versus the control group.

Lipid peroxidation is directly associated with elevated ROS levels, as observed in our study. A 2.5-fold increase (*** *p* ≤ 0.001) in MDA levels was observed in the hypothalamus (Figure 2A) and striatum (Figure 2C) with ipconazole at a dose of 20 mg/kg b.w. Similarly, MDA levels increased dose-dependently with ipconazole concentrations of 5, 10 and 20 mg/kg b.w. in the cerebral cortex (1.3-, 1.5- and 1.5-fold, respectively) (Figure 2B) and hippocampus (1.6-, 2- and 3-fold, respectively) (Figure 2D).

### 3.3. Catalase Activity 

**Figure 3:**
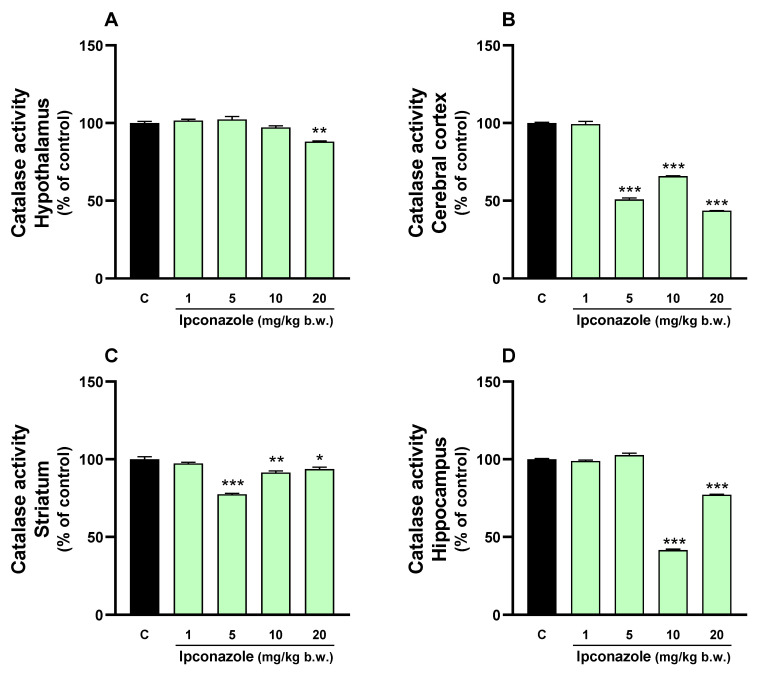
Catalase activity was measured in the (**A**) hypothalamus, (**B**) cerebral cortex, (**C**) striatum and (**D**) hippocampus of rat brains. Results are presented as a percentage of the control (mean ± SEM) based on four replicates per treatment. The significance between the treatments (green bars) and the control group (black bar) was determined by one-way ANOVA using Tukey’s post hoc test. * *p* ≤ 0.05, ** *p* ≤ 0.01 and *** *p* ≤ 0.001 indicate significant differences between the treatments versus the control group.

Catalase oxidoreductase enzyme activity was evaluated in the brain regions of albino rats exposed to ipconazole at doses of 1, 5, 10 and 20 mg/kg b.w. for 6 days. A decrease in catalase enzyme activity was observed in all brain regions because of ipconazole exposure. In the hypothalamus (Figure 3A), catalase activity was reduced by 12% (** *p* ≤ 0.01) at the 20 mg/kg b.w. dose of ipconazole. In the cerebral cortex (Figure 3B), there was a significant (*** *p* ≤ 0.001) reduction in catalase enzyme activity by 50% (5 mg/kg b.w.), 35% (10 mg/kg b.w.) and 57% (20 mg/kg b.w.). In the striatum (Figure 3C), catalase enzyme activity was significantly decreased by 23% (*** *p* ≤ 0.001) at 5 mg/kg b.w., 9% (** *p* ≤ 0.01) at 10 mg/kg b.w. and 7% (*p ≤ 0.05) at 20 mg/kg b.w. Finally, in the hippocampus (Figure 3D), there was a significant reduction (*** *p* ≤ 0.001) in catalase enzyme activity by 58% at 10 mg/kg b.w. and 23% at 20 mg/kg b.w. of ipconazole.

### 3.4. Molecular Biomarkers of Cell Death

**Figure 4:**
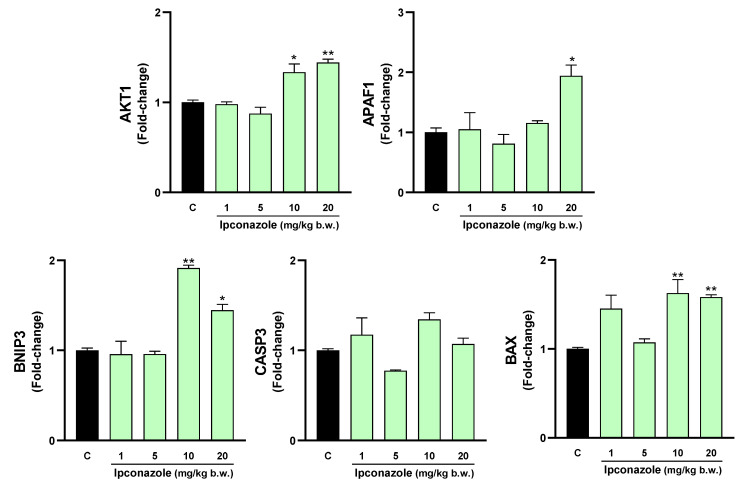
Real-time molecular expression of cell death biomarkers (AKT1, APAF1, BNIP3, CASP3 and BAX) in the rat hypothalamus. Results are presented as mean ± SEM of four replicates per treatment. The significance between the treatments (green bars) and the control group (black bar) was determined by one-way ANOVA using Tukey’s post hoc test. * *p* ≤ 0.05 and ** *p* ≤ 0.01 indicate the significance between the treatments versus the control group.

Increased oxidative stress products, such as ROS and MDA, and a possible decrease in antioxidant activity can trigger cell death. In our study, we evaluated the molecular expression of cell death-related biomarkers in the brain regions of rats exposed to different concentrations of ipconazole. As shown in Figure 4, ipconazole at doses of 10 and 20 mg/kg b.w. produced a significant increase in the molecular biomarkers AKT1 (1.3- and 1.4-fold, respectively), BNIP3 (1.9- and 1.4-fold, respectively) and BAX (1.6- and 1.6-fold, respectively). Additionally, ipconazole at a dose of 20 mg/kg b.w. produced a significant 1.9-fold increase in the biomarker APAF1. 

**Figure 5:**
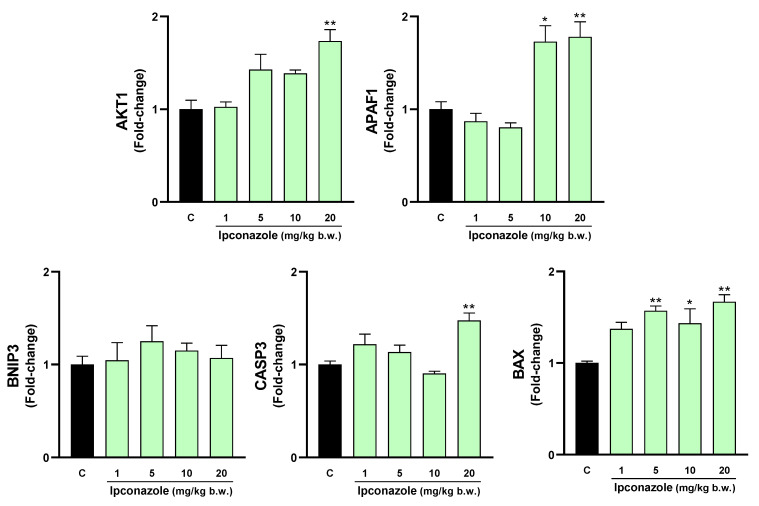
Real-time molecular expression of cell death biomarkers (AKT1, APAF1, BNIP3, CASP3 and BAX) in the rat cerebral cortex. Results are presented as mean ± SEM of four replicates per treatment. The significance between the treatments (green bars) and the control group (black bar) was determined by one-way ANOVA using Tukey’s post hoc test. * *p* ≤ 0.05 and ** *p* ≤ 0.01 indicate the significance between the treatments versus the control group.

Figure 5 shows that in the cerebral cortex, ipconazole at doses of 5, 10 and 20 mg/kg b.w. increased the expression of the molecular biomarker BAX by 1.6-, 1.4- and 1.7-fold, respectively, in a dose-dependent manner relative to the control. Furthermore, ipconazole at doses of 10 and 20 mg/kg b.w. resulted in a 1.7- and 1.8-fold increase in the biomarker APAF1. At 20 mg/kg b.w., the AKT1 and CASP3 biomarkers increased 1.7- and 1.5-fold, respectively, compared to the control. 

**Figure 6:**
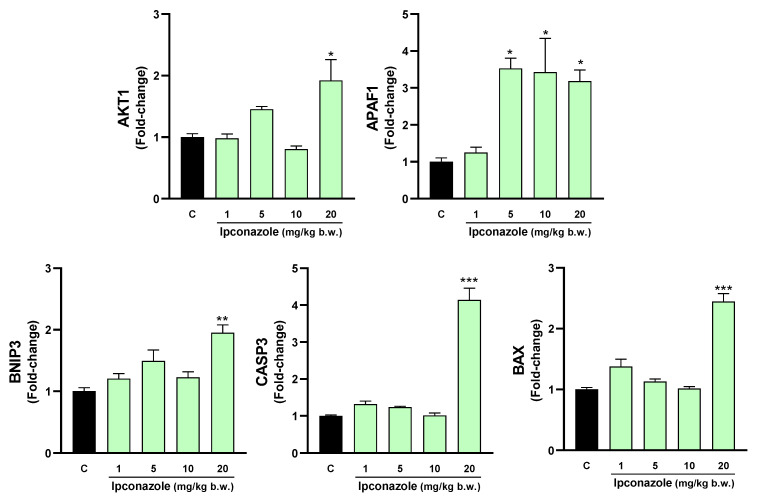
Real-time molecular expression of cell death biomarkers (AKT1, APAF1, BNIP3, CASP3 and BAX) in the rat striatum. Results are presented as mean ± SEM of four replicates per treatment. The significance between the treatments (green bars) and the control group (black bar) was determined by one-way ANOVA using Tukey’s post hoc test. * *p* ≤ 0.05, ** *p* ≤ 0.01 and *** *p* ≤ 0.001 indicate the significance between the treatments versus the control group.

In the striatum, ipconazole increased APAF1 molecular expression in a dose-dependent manner by 3.5- (5 mg/kg b.w.), 3.4- (10 mg/kg b.w.) and 3.1-fold (20 mg/kg b.w.), compared to the control. Furthermore, at the highest dose of ipconazole (20 mg/kg b.w.), the molecular expression of AKT1 (1.9-fold), BNIP3 (1.9-fold), CASP3 (4.1-fold) and BAX (2.4-fold) was increased (Figure 6).

**Figure 7:**
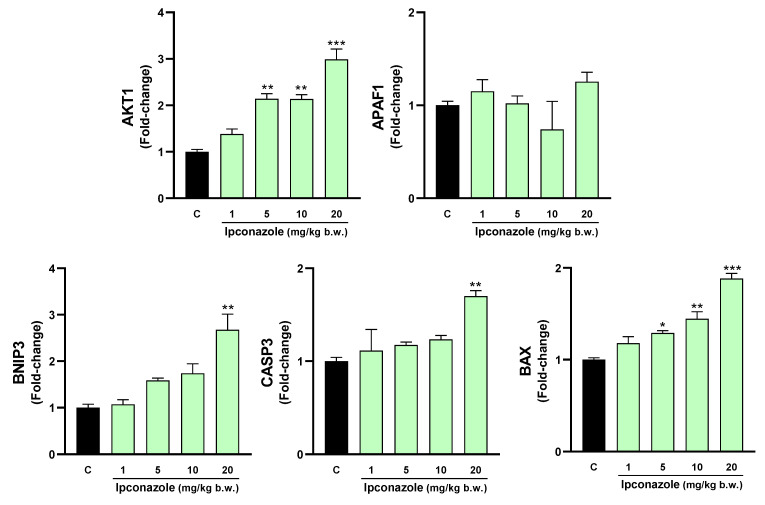
Real-time molecular expression of cell death biomarkers (AKT1, APAF1, BNIP3, CASP3 and BAX) in the rat hippocampus. Results are presented as mean ± SEM of four replicates per treatment. The significance between the treatments (green bars) and the control group (black bar) was determined by one-way ANOVA using Tukey’s post hoc test. * *p* ≤ 0.05, ** *p* ≤ 0.01 and *** *p* ≤ 0.001 indicate the significance between the treatments versus the control group.

In the hippocampus (Figure 7), ipconazole at doses of 5, 10 and 20 mg/kg b.w. increased the expression levels of AKT1 (2.1-, 2.1- and 2.9-fold, respectively) and BAX (1.3-, 1.4- and 1.8-fold, respectively). The highest dose of ipconazole produced increases in the molecular expression of BNIP3 (2.7-fold) and CASP3 (1.7-fold), compared to the control.

### 3.5. Molecular Biomarkers Related to the Inflammasome Complex

**Figure 8:**
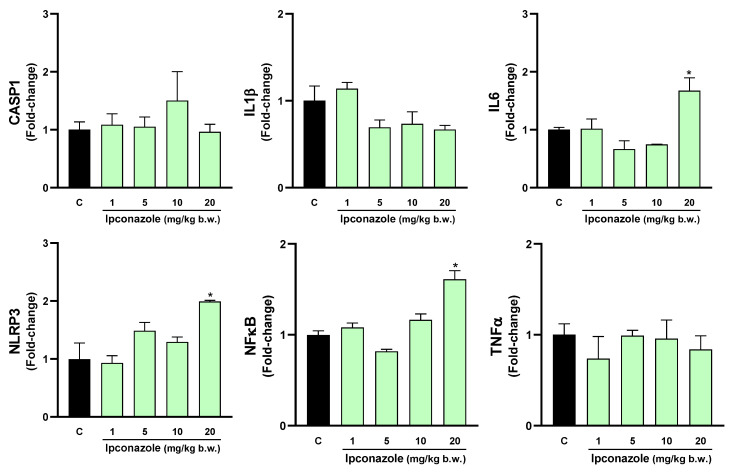
Real-time molecular expression of biomarkers related to the inflammasome complex (CASP1, IL1β, IL6, NLRP3, NFκB and TNFα) in the rat hypothalamus. Results are presented as mean ± SEM of four replicates per treatment. The significance between the treatments (green bars) and the control group (black bar) was determined by one-way ANOVA using Tukey’s post hoc test. * *p* ≤ 0.05 indicates the significance between the treatments versus the control group.

**Figure 9:**
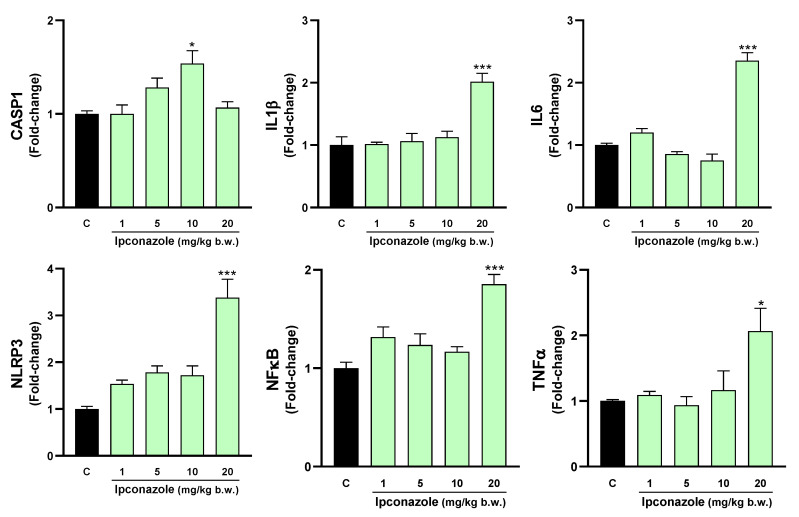
Real-time molecular expression of biomarkers related to the inflammasome complex (CASP1, IL1β, IL6, NLRP3, NFκB and TNFα) in the rat cerebral cortex. Results are presented as mean ± SEM of four replicates per treatment. The significance between the treatments (green bars) and the control group (black bar) was determined by one-way ANOVA using Tukey’s post hoc test. * *p* ≤ 0.05 and *** *p* ≤ 0.001 indicate the significance between the treatments versus the control group.

**Figure 10:**
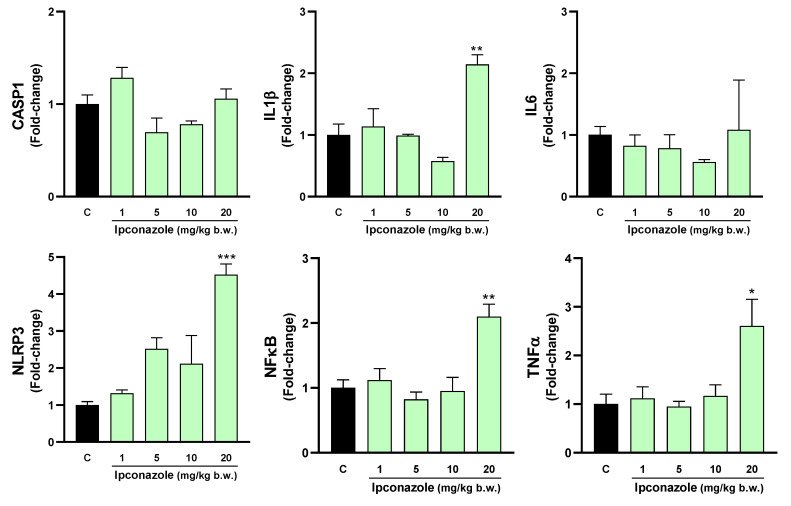
Real-time molecular expression of biomarkers related to the inflammasome complex (CASP1, IL1β, IL6, NLRP3, NFκB and TNFα) in the rat striatum. Results are presented as mean ± SEM of four replicates per treatment. The significance between the treatments (green bars) and the control group (black bar) was determined by one-way ANOVA using Tukey’s post hoc test. * *p* ≤ 0.05, ** *p* ≤ 0.01 and *** *p* ≤ 0.001 indicate the significance between the treatments versus the control group.

**Figure 11:**
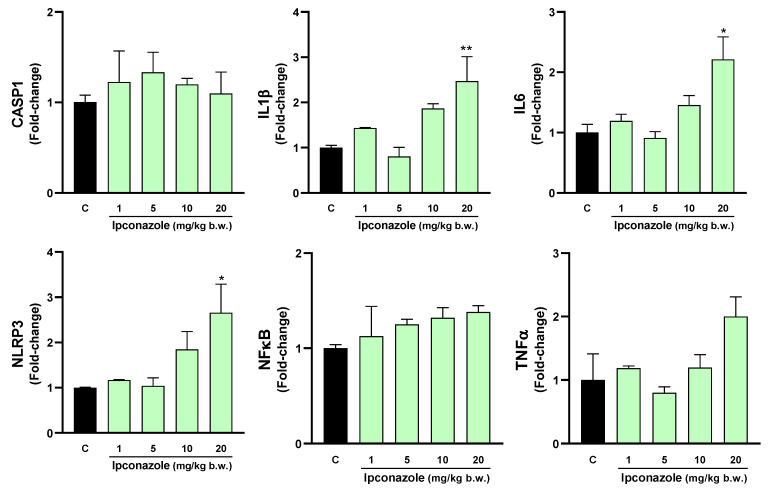
Real-time molecular expression of biomarkers related to the inflammasome complex (CASP1, IL1β, IL6, NLRP3, NFκB and TNFα) in the rat hippocampus. Results are presented as mean ± SEM of four replicates per treatment. The significance between the treatments (green bars) and the control group (black bar) was determined by one-way ANOVA using Tukey’s post hoc test. * *p* ≤ 0.05 and ** *p* ≤ 0.01 indicate the significance between the treatments versus the control group.

The inflammasome is a cytosolic multiprotein complex that triggers the activation of a strong inflammatory response, and its activation may be strongly related to oxidative stress. In this study, we evaluated the effect of ipconazole on the molecular expression of mediators directly and indirectly related to the formation of the inflammasome complex. In the hypothalamus, ipconazole at a dose of 20 mg/kg b.w. significantly increased the molecular expression of the biomarkers IL6 (1.7-fold), NLRP3 (2-fold) and NFκB (1.6-fold), compared to the control (Figure 8). In the cerebral cortex, the dose of 10 mg/kg b.w. of ipconazole increased the molecular expression of the biomarker CASP1 by 1.5-fold, while the dose of 20 mg/kg b.w. of ipconazole increased the molecular expressions of IL1β, IL6, NLRP3, NFκB and TNFα by 2-, 2.4-, 3.4-, 1.9- and 2-fold, respectively (Figure 9). Similar effects of the fungicide ipconazole (at a dose of 20 mg/kg b.w.) were observed in the striatum (Figure 10) and hippocampus (Figure 11), with significant increases in the expression of IL1β (2.1-fold), NLRP3 (4.5-fold), NFκB (2.1-fold) and TNFα (2.6-fold) (Figure 10) and IL1β (2.5-fold), IL6 (2.2-fold) and NLRP3 (2.6-fold) (Figure 11).

## 4. Discussion

Several studies have shown that triazole fungicides can be absorbed through the skin, gastrointestinal tract and pulmonary tract and can even easily cross the blood–brain barrier and placental barrier, causing severe damage to the body [30]. In our study, experimental animals were orally exposed to different doses of the fungicide ipconazole to assess its toxicity in brain regions including the hypothalamus, cerebral cortex, striatum and hippocampus.

In the present study, ipconazole significantly increased ROS production in brain regions in a dose-dependent manner at doses of 5, 10 and 20 mg/kg b.w., indicating the oxidative action of this pesticide. Similarly, MDA levels increased with ipconazole at the highest dose in the hypothalamus and striatum, and in a dose-dependent manner in the cerebral cortex and hippocampus. These results demonstrate a close relationship between the high levels of ROS and MDA due to ipconazole exposure. The increase in ROS arises from an imbalance between antioxidant and prooxidant molecules, usually induced by endogenous or exogenous agents, leading to the activation of signaling pathways associated with cell damage or the production of other more dangerous prooxidant molecules such as MDA [31]. ROS are the main effector molecules of oxidative stress, occurring in physiological or pathological states, and are produced in mitochondria and cellular endomembrane where enzymatic reactions and autooxidation of various compounds occur [32]. Many exogenous factors, such as pesticide chemicals, precipitate ROS production, which in turn produces other free radicals or metabolites from lipid peroxidation such as MDA. MDA is formed from the reaction of free radicals and lipids and can alter cell membrane structure, subsequently causing DNA alterations at the cellular level [33]. Polyunsaturated fatty acids are the biomolecules most affected by oxidative stress, leading to lipid peroxidation, the main product of which is MDA, known for its mutagenic and toxic effects [34].

Several in vivo and in vitro studies have shown that pesticides can induce oxidative stress in the nervous system through the production of ROS and MDA, as reported in the present study. In the case of triazole fungicides, we have previously described the oxidative cytotoxic effect (increased ROS production) of ipconazole on SH-SY5Y neuronal cells [18]. Similar results have also been described with other triazoles. For instance, the fungicide epoxiconazole induced an increase of ROS and MDA in rat PC12 cells [35], while the triazoles propiconazole and tebuconazole at high doses induced a significant increase in ROS and MDA in SH-SY5Y cells [36]. Additionally, a mixture of six triazoles (imazalil, flusilazole, fluconazole, tebuconazole, triadimefon and cyproconazole) had a low effect on ROS production in PC12 cells [37]. Furthermore, penconazole increased MDA levels in rat brains [38], and the fungicide difenoconazole augmented ROS levels in carp brains [39].

The effective way to reduce the deleterious effects of cellular oxidative stress is to increase the activity of antioxidant enzymes. In the present study, we evaluated the effect of ipconazole on the activity of the enzyme catalase, which is capable of converting hydrogen peroxide (H_2_O_2_) into water and oxygen. In the hypothalamus, only the highest dose of ipconazole resulted in a significant decrease in catalase enzyme activity, while ipconazole dose-dependently decreased catalase activity in the other brain regions studied. Lower catalase activity could lead to an increase in H_2_O_2_, which at high concentrations is toxic because it can easily be converted into the superoxide anion (O_2_•−) and hydroxyl radical (•OH), molecules closely related to oxidative damage and neuronal aging. In addition, brain catalase levels are lower compared to other organs, requiring the action of peroxidases for H_2_O_2_ removal [40,41]. Similar effects to those observed in our study have been described for the fungicide penconazole, which decreased catalase enzyme activity in rat brains [38]. This effect was also observed in the nervous system of carp after exposure to difenoconazole [39]. Conversely, the fungicide propiconazole increased catalase activity in zebrafish brains [8], and epoxiconazole increased catalase activity 8-fold in rat F98 glial cells [42]. 

The results presented indicate that ipconazole could negatively alter the oxidative status in the rat brain by increasing the production of ROS and MDA while decreasing the activity of the enzyme catalase. Elevated levels of free radicals can activate cellular pathways that lead to increased cell damage and even cell death. Increased ROS serves as a cell death-inducing factor when it activates the intrinsic DNA damage apoptosis pathway. These stress signals typically activate proapoptotic proteins such as BAX, BNIP3, APAF1 and CASP3 [42,43,44,45]. 

The present study demonstrates that the fungicide ipconazole increased the molecular expression of several biomarkers (APAF1, BNIP3, CASP3 and BAX) of cell death in all brain regions studied (hypothalamus, cerebral cortex, striatum and hippocampus). This increase in the expression of cell death biomarkers could be attributed to the elevated levels of ROS and MDA found in this study. Other studies have shown similar results regarding the effect of triazoles. For instance, penconazole up-regulated BAX and CASP3 mRNA in rat brains [46]; ipconazole induced molecular expression of BAX, CASP3, APAF1 and BNIP3 and increased caspase 3/7 enzyme activity in SH-SY5Y cells [18]; and epoxiconazole induced apoptosis in rat PC12 cells [35]. The overexpression of various cell death biomarkers such as APAF1 would be associated with neurodegenerative pathologies related to neuronal death [47] by acting as proapoptotic proteins and producing mitochondrial dysfunction, which is the case of BNIP3 [48]; these neurodegenerative effects were demonstrated in dopaminergic neuronal death following BAX overexpression [49]. Furthermore, caspase-3 overexpression in Alzheimer’s disease confirms the role of the extrinsic pathway in neuronal apoptosis and amyloid-β deposition [50].

ROS are a group of molecules including H_2_O_2_, O_2_•− and •OH, which play roles in maintaining redox homeostasis at physiological levels [51]. However, their excess can lead to deleterious effects by activating inflammatory pathways such as the inflammasome complex associated with apoptosis [52]. The NLRP3 inflammasome complex serves as a cellular stress sensor, as its activation depends on ROS generation [53,54]. Mitochondrial dysfunction, characterized by DNA damage and excessive ROS production, contributes to NLRP3 inflammasome activation. This activation triggers CASP1 activation and the release of the cytokine IL1β in response to cellular damage signals associated with mitochondrial injury [55,56]. It is known that oxidized mitochondrial DNA (OX-mtDNA) can induce NLRP3 inflammasome activation during apoptosis, and that this OX-mtDNA is released from mitochondria due to NFκB-induced mitochondrial dysfunction in the presence of ATP. Subsequently, OX-mtDNA binds to and activates the NLRP3 inflammasome in the cytoplasm [57]. 

Furthermore, ROS not only directly regulate the inflammasome assembly process but also indirectly regulate inflammasome activity by affecting cytoplasmic proteins [58]. ROS-induced activation of the NFκB pathway not only stimulates NLRP3 inflammasome formation but also directly promotes the expression of TNFα, pro-IL1β, IL6 and other components of the inflammasome complex [59]. Activation or overexpression of the inflammasome complex has been reported in diseases such as Alzheimer’s or Parkinson’s disease. In post mortem studies of brains from Parkinson’s or Alzheimer’s patients, overexpression of IL1β and Casp1 was reported [60]. Fibrillar amyloid-β was also shown to act as a trigger for NLRP3 microglial inflammasome assembly or NFκB response [61,62].

It is plausible that the results of our study are consistent with the above findings, as several biomarkers (CASP1, IL1β, IL6, NLRP3, NFκB and TNFα) related to the inflammasome complex showed increased molecular expression in the hypothalamus, cerebral cortex, striatum and hippocampus of rats exposed to the fungicide ipconazole. A similar effect of ipconazole has been reported in SH-SY5Y cells [18].

## 5. Conclusions

In conclusion, a six-day acute oral exposure to the fungicide ipconazole in rats resulted in increased production of ROS and MDA, accompanied by decreased activity of the antioxidant enzyme catalase. These changes exacerbated oxidative stress across all brain regions studied. Additionally, this oxidative damage likely contributed to the up-regulation of molecular biomarkers associated with cell death (AKT1, APAF1, BNIP3, CASP3 and BAX) and inflammasome complex (CASP1, IL1β, IL6, NLRP3, NFĸB and TNFα) activation. Our findings suggest that ipconazole should be considered an environmental risk factor for the development of nervous system disorders in mammals.

## Data Availability

Data are contained within the article.

## Supplementary Materials

The following supporting information can be downloaded at: https://www.mdpi.com/article/10.3390/toxics12090638/s1, S1: Forward and reverse sequences for genes related to cell death and inflammasome complex biomarkers.
