# Supplementary material for: The Fungicide Ipconazole Can Activate Mediators of Cellular Damage in Rat Brain Regions
Source: Toxics. 2024 Aug 31;12(9):638. doi: 10.3390/toxics12090638 (PMC11435560; doi:10.3390/toxics12090638)
Supplement: Supplementary file 1 [file toxics-12-00638-s001.zip › toxics-3180190-supplementary.pdf]

Supplementary materials

S1. Forward and reverse sequences for genes related to cell death and inflammasome complex biomarkers.

Gene	Forward	Reverse
Cell death		
BAX (Bcl-2-associated X protein)	'CTGCAGAGGATGATTGCTGA'	'GATCAGCTCGGGCACTTTAG'
CASP3 (Caspase 3)	'AATTCAAGGGACGGGTCATG'	'GCTTGTGCGCGTACAGTTTC'
APAF1 (Apoptotic protease-activating factor 1)	'TTCAGGTTTGTAGCTCGGCA'	'ACCCAAGGATCCCAAACGTC'
BNIP3 (BCL2-interacting protein 3)	'TTTTAAACACCCGAAGCGCA'	'TGAGCAGAAGGCAGATCCAA'
AKT1 (AKT serine/threonine kinase 1)	'CACCGCTTCTTTGCCAACAT'	'CACACACTCCATGCTGTCATCT'
Inflammasome complex		
NLRP3 (NLR pyrin domain containing 3)	'CTGCATGCCGTATCTGGTTG '	'GCTGAGCAAGCTAAAGGCTTC'
CASP1 (Caspase 1)	'TGGAGCTTCAGTCAGGTCCAT'	'ATGCGCCACCTTCTTTGTTC'
IL1β (Interleukin-1 beta)	'TGGCAACTGTCCCTGAACTC'	'GTCGAGATGCTGCTGTGAGA'
NFκB (Nuclear factor kappa B)	'ATATTCACCTGCACGCCCAC'	'GGTTTGCAAAGCCAACCACC '
TNFα (Tumor necrosis factor alpha)	'ATCCGAGATGTGGAACTGGC'	'AAATGGCAAATCGGCTGACG'
IL6 (Interleukin 6)	'CACTTCACAAGTCGGAGGCT'	'AGCACACTAGGTTTGCCGAG'
Normalizer		
GAPDH (glyceraldehyde-3-phosphate dehydrogenase)	'TCCCTGTTCTAGAGACAG'	'CCACTTTGTCACAAGAGA'
